# VEB-1 Extended-Spectrum β-lactamase–producing *Acinetobacter baumannii*, France^1^

**DOI:** 10.3201/eid1208.051547

**Published:** 2006-08

**Authors:** Thierry Naas, Bruno Coignard, Anne Carbonne, Karine Blanckaert, Odile Bajolet, Claude Bernet, Xavier Verdeil, Pascal Astagneau, Jean-Claude Desenclos, Patrice Nordmann, on behalf of, the French Nosocomial Infection Early Warning, Investigation and Surveillance Network

**Affiliations:** * Hôpital de Bicêtre, Le Kremlin-Bicêtre, France;; † Institut de Veille Sanitaire, Saint-Maurice, France;; ‡ Centre de Coordination de Lutte contre les Infections Nosocomiales Paris Nord, Paris, France;; § Réseau Bactéries Multi-Résistantes Champagne-Ardennes, Centre Hospitalier et Universitaire, Reims, France;; ¶ Centre de Coordination de Lutte contre les Infections Nosocomiales Sud-Est, Lyon, France;; # Centre de Coordination de Lutte Contre les Infections Nosocomiales Sud-Ouest et Centre Hospitalier et Universitaire, Toulouse, France

**Keywords:** *A. baumannii*, ESBL, VEB-1, outbreak, France, mandatory notification, research

## Abstract

VEB-1 extended-spectrum β-lactamase–producing *Acinetobacter baumannii* was responsible for an outbreak in hospitals in France. A national alert was triggered in September 2003 when 4 hospitals reported clusters of *A. baumannii* infection with similar susceptibility profiles. Case definitions and laboratory guidelines were disseminated, and prospective surveillance was implemented; strains were sent to a single laboratory for characterization and typing. From April 2003 through June 2004, 53 hospitals reported 290 cases of *A. baumannii* infection or colonization; 275 isolates were *bla*_VEB-1_-positive and clonally related. Cases were first reported in 5 districts of northern France, then in 10 other districts in 4 regions. Within a region, interhospital spread was associated with patient transfer. In northern France, investigation and control measures led to a reduction of reported cases after January 2004. The national alert enabled early control of new clusters, demonstrating the usefulness of early warning about antimicrobial drug resistance.

During the past decade, nosocomial outbreaks of *Acinetobacter baumannii* have been described with increasing frequency, occurring mostly in intensive care units, burn units, and surgical wards (*1**,**2*). Epidemic strains of *A. baumannii* are often resistant to several antimicrobial drugs, which reduces treatment effectiveness. Nosocomial transmission is from patient to patient and associated with environmental reservoirs (*2*). Several risk factors for *A. baumannii* infections have been identified and include severity of underlying disease, duration of hospitalization, invasive procedures, and prior broad-spectrum antimicrobial drug therapy (*2**–**4*). *A. baumannii* can be detected in patients without infection (i.e., colonization) or as the source in patients with severe infections; the case-fatality ratio varies from 17% to 46% for septicemia and can be as high as 70% for pneumonia (*1*).

*A. baumannii* is not the most common antimicrobial drug–resistant pathogen in hospitalized patients; it accounted for 1.2% of all nosocomial infections in 2001 in France (*5*). However, increasing therapeutic difficulty caused by resistance is a serious concern (*6**–**9*). A variety of molecular mechanisms for resistance to broad-spectrum β-lactams have been reported in *A. baumannii*, such as mutations of penicillin-binding proteins and alterations of membrane permeability, but the most common mechanism is attributed to the presence of β-lactamases encoded by either chromosomes or plasmids (*2**,**10**,**11*). Several class A, B, and D β-lactamases (*2**,**8*) as well as chromosome-mediated cephalosporinases (*12*) confer various resistance phenotypes. Moreover, extended-spectrum β-lactamase (ESBL)–producing *A. baumannii* strains have also been described: PER-1 in Turkey, Korea, and France (*13**–**15*); VEB-1 in France (*4**,**16*); and CTX-M-2 recently in Japan (*17*).

The *bla*_VEB-1_ ESBL gene is located in a class 1 integron initially detected in *Enterobacteriaceae* and *Pseudomonas aeruginosa* from Southeast Asia (*18**–**20*). Subsequently, it has been described in clonally related *A. baumannii* isolates recovered during an outbreak that lasted 9 months (August 2001–April 2002) in the intensive care unit of a hospital in northern France (*4**,**16*). In these strains, the location of the *bla*_VEB-1_ gene on the chromosomes and integrons was identified (*4**,**16*). One year after this outbreak was controlled, nosocomial infections with this *A. baumannii* strain reemerged in the same area and subsequently spread to hospitals located in other districts in France. We describe the nationwide spread of this strain from April 2003 through June 2004.

In early September 2003, an alert was triggered through the national nosocomial infection notification system when, within a month, 4 hospitals in a single district (Nord) reported 5 clusters of *A. baumannii* infections with a similar susceptibility profile; all *A. baumannii* strains were confirmed positive for VEB-1. In October 2003, the National Institute of Public Health (Institut de Veille Sanitaire [InVS]) alerted all hospitals in France of the emergence of this VEB-1–producing *A. baumannii* strain, disseminated case definitions and laboratory guidelines, and implemented a prospective, laboratory-based national surveillance system.

## Materials and Methods

### Case Definitions

A probable case was defined as follows: isolation of an *A. baumannii* strain showing a multidrug-resistance profile similar to that of the 2001 outbreak strain, susceptible to only imipenem and colistin (strain AYE [16]), from a patient hospitalized in France between April 1, 2003, and June 30, 2004; only 1 isolate per patient was retained for the study period. A confirmed case was defined as a case for which VEB-1 ESBL production had been confirmed by the central laboratory, which used phenotypic (detection of the synergy image) and genotypic (PCR amplification of the *bla*_VEB-1_ gene) methods. Infection or colonization was ascertained by clinicians according to national case definitions for nosocomial infections adapted from the Centers for Disease Control and Prevention (*21*).

### Epidemiologic Investigation

Case definitions were dispatched to hospital laboratories and infection control units. Cases had to be reported to regional infection control coordinating centers (CCLIN) (*22*) and local health departments through the national nosocomial infection notification system. In this system, implemented in August 2001, baseline reporting requirements use specific criteria; 1 of them is about rare microorganisms, depending on virulence and antimicrobial drug susceptibility (*23*). No list of microorganisms or resistance phenotypes exists; reports are based on the epidemiologic knowledge of the infection control units. Hospitals report >1 nosocomial infections on a simple form, which summarizes cases and investigations and allows hospitals to request assistance when needed. For the purpose of this investigation, this system was reinforced as hospitals were asked to report not only *A. baumannii* infections but also instances of colonization and to send bacterial strains to a central laboratory. All reported cases were investigated by infection control units, local health departments, and CCLIN, the latter offering on-site assistance to hospitals when needed. Data on all cases were centralized and analyzed by InVS, which coordinated the investigation through the Nosocomial Infection Early Warning, Investigation and Surveillance Network (Réseau d'Alerte, d'Investigation et de Surveillance des Infections Nosocomiale [RAISIN]), a partnership between InVS and CCLIN.

### Microbiologic Investigation

All isolates of *A. baumannii* were recovered from routine clinical specimens (from blood and catheters, urine, respiratory tract, skin, and wounds) and from colonization samples (from axillary, pharyngeal, or rectal swabs), identified by standard techniques at local laboratories, then sent for confirmatory testing to a central laboratory (University Hospital of Bicêtre, K.-Bicêtre, France).

Identification was confirmed by using the API 32GN system (bioMérieux, Marcy-l'Etoile, France). *A. baumannii* strains were also tested for the ability to grow at 44°C in Trypticase soy broth (Oxoid, Unipath Ltd, Basingstoke, UK).

Routine antibiograms were determined by the disk diffusion method on Mueller-Hinton agar (BioRad, Marnes-La-Coquette, France) and interpreted as recommended by the Clinical and Laboratory Standards Institute (formerly NCCLS) (*24*). The presence of ESBL was shown by a synergy image created by using the double-disk synergy test with cefepime, ceftazidime, and ticarcillin-clavulanic acid disks on Mueller-Hinton agar plates (*16**,**18*). Synergy images were best seen when plates were incubated at 25°C. Alternatively, double-disk synergy tests were also performed on plates containing cloxacillin (200 μg/mL) (*16*). Analytic isoelectric focusing was performed with an ampholine polyacrylamide gel (*18*).

### Molecular Investigation

Genomic DNA and plasmid extractions and electroporation of plasmid extracts into *Escherichia coli* DH10B were performed (*16*). Half of *A. baumannii* isolates from the 4 largest hospitals of 2 districts were randomly selected during the outbreak; all isolates from smaller hospitals or from other districts were analyzed by pulsed-field gel electrophoresis (PFGE), using *Apa*I (Amersham Biosciences, Les Ulis, France) (*4*). *Apa*I-macrorestriction patterns were digitized and analyzed with Taxotron software (Institut Pasteur, Paris, France) and interpreted according to Tenover et al. (*25*).

PCR-based amplification of class 1 integron structures and of *bla*_VEB-1_ gene and subsequent sequencing of the *bla*_VEB-1_ gene were performed as described (*19*). Automated sequencing reactions were performed with the same *bla*_VEB-1_-specific primers (ABI Prism 3100; Applied Biosystems, Les Ullis, France).

## Results

### Epidemiologic Investigation

**Figure 1:**
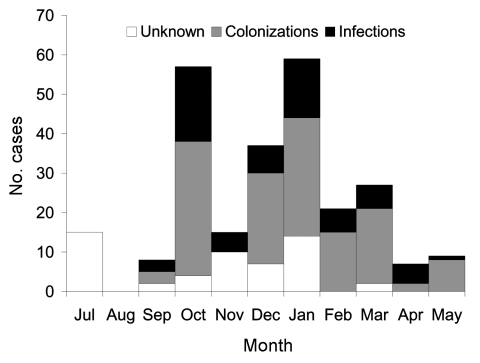
Number of VEB-1–producing *Acinetobacter baumannii* cases, by month of report, France, July 2003–May 2004 (N = 255).

From April 1, 2004, to June 30, 2004, 53 hospitals (41 tertiary care and 12 long-term care facilities) located in 15 districts reported 290 probable cases, of which 275 (95%) were confirmed. Of the 290 probable cases, 255 (88%) were reported through 116 mandatory notifications, and 35 (12%) were identified through strains directly sent for characterization without notification. The 2 first notifications occurred at the end of July (2 clusters totaling 15 cases) and were followed by 3 other notifications in early September (3 clusters totaling 8 cases). The monthly number of reported cases peaked in October 2003 and again in January 2004 (after intense media coverage of the outbreak); it gradually declined after this date, until the alert was canceled in June 2004 (Figure 1).

**Figure 2:**
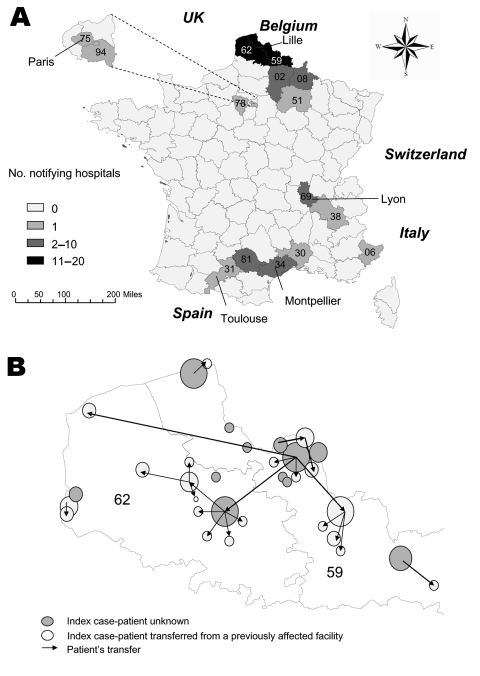
A) Hospitals reporting VEB-1–producing *Acinetobacter baumannii*, by district, France, April 2003–May 2004 (N = 53). Each district is identified with a number (same numbers used in Table 1). B) Interhospital spread in northern France. Circles represent affected hospitals; the sizes are proportional to the number of reported cases.

Cases were first reported in the Nord district and in 4 other contiguous districts, then later in 10 districts in 4 noncontiguous regions. Most reporting hospitals and cases were in 2 adjacent districts (59 and 62 on Figure 2A). The date of case diagnosis indicated that the strain had been circulating since April 2003 in 5 districts of northern France before the outbreak was recognized (Table 1).

**Table 1:** VEB-1–producing *Acinetobacter baumannii* case characteristics and period of transmission, by district, France, April 2003–May 2004 (N = 290)

District*	Reporting hospitals (n)	Reported cases (n)†				Reported deaths (n)	Date of case diagnosis	
		Total	I	C	U		First	Last
Nord (59)	19	124	19	60	45	24	22 Apr 2003	13 May 2004
Pas de Calais (62)	14	111	32	55	24	7	9 Mar 2003	14 May 2004
Aisne (02)	2	2	0	0	2	0	5 Nov 2003	5 Nov 2003
Ardennes (08)	2	14	6	8	0	0	15 May 2003	2 Feb 2004
Champagne (51)	1	11	2	9	0	1	13 Aug 2003	15 Mar 2004
Paris (75)	1	1	1	0	0	0	31 Jan 2004	31 Jan 2004
Val de Marne (94)	1	1	1	0	0	0	3 May 2004	3 May 2004
Yvelines (78)	1	1	0	1	0	0	8 Mar 2004	8 Mar 2004
Rhône (69)	4	8	4	4	0	0	13 Aug 2003	7 Feb 2004
Isère (38)	1	1	0	1	0	0	2 Dec 2003	2 Dec 2003
Haute Garonne (31)	1	5	1	2	2	1	16 Sep 2003	15 Apr 2004
Tarn (81)	2	2	1	1	0	0	28 Oct 2003	5 Jan 2004
Hérault (34)	2	7	5	2	0	1	21 Oct 2003	9 Apr 2004
Gard (30)	1	1	1	0	0	0	10 Apr 2004	10 Apr 2004
Alpes Maritimes (06)	1	1	0	1	0	0	3 Jan 2004	3 Jan 2004
Total	53	290	73	144	73	34	22 Apr 2003	14 May 2004

*Districts are listed from north to south and west to east; numbers are those used in Figure 2 to identify districts. †I, infected; C, colonized; U, unknown.

Spread of this multidrug-resistant strain was mediated by large referring hospitals. Among 116 notifications, 20% came from regional teaching hospitals, 45% from public general hospitals, 15% from smaller private hospitals, and 20% from long-term care facilities; most affected wards were intensive care units (54 notifications, 47%), medical wards (55 notifications, 47%), reeducation and long-term care wards (24 notifications, 21%), and surgical wards (13 notifications, 11%). In 3 regions (northern France, Toulouse-Montpellier, Lyon), investigations suggested that frequent patient transfers between hospitals within the same healthcare network could explain the diffusion of the strain (*4**,**26*). In northern France (Nord and Pas de Calais districts), among 33 hospitals reporting >1 cases, 22 (67%) had an index case directly admitted from a previously affected facility (Figure 2B); however, no such link could be established from 1 region to another.

Of the 217 (71%) cases with clinical documentation, 73 (33%) were infections and 144 (67%) were colonizations. The sources of the clinical isolates were as follows: respiratory tract (33%), skin and wounds (33%), urine (21%), catheters and blood (8%), and others (5%). By the time of notification, 34 (12%) patients had died; however, investigations in northern France suggested that only 17% of the reported deaths were related to the *A. baumannii* infection.

At several participating hospitals, environmental surfaces were swabbed for culture and found to be positive for VEB-1-producing *A. baumannii*; the organism was particularly prevalent on bed rails and respiratory equipment (data not shown) and, at 1 hospital, was also found on blood pressure cuffs (*27*).

### Microbiologic Investigation

**Figure 3:**
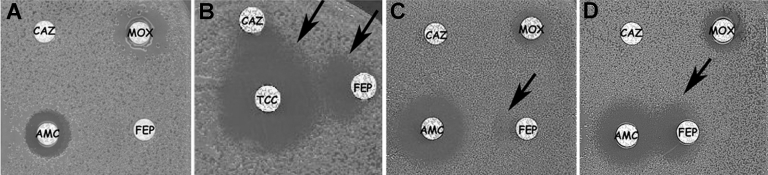
Extended-spectrum β-lactamase (ESBL) laboratory identification. Usefulness of double-disk synergy test with *bla*_VEB-1_-positive *Acinetobacter baumannii* strain on Mueller-Hinton agar plates with clavulanate as inhibitor. The disks tested contained ticarcillin + clavulanate (TCC), amoxicillin + clavulanate (AMC), moxalactam (MOX), ceftazidime (CAZ), and cefepime (FEP). A) Standard disk diffusion as recommended by Clinical and Laboratory Standards Institute at 37°C (98°F). B) Standard disk diffusion on cloxacillin-containing Mueller-Hinton plates at 37°C (98°F). Cloxacillin inhibits partially the naturally occurring cephalosporinase (AmpC) from *A. baumannii*, thus enabling easier detection of possible ESBL phenotypes. C) Standard disk diffusion at 25°C (77°F). D) Standard disk diffusion at 25°C (77°F) when AMC and FEP disks were brought closer. The presence of ESBL was shown by a synergy image, as indicated with the arrows. ESBL presence was best seen on cloxacillin-containing (B) plates or at reduced growth temperature (D).

The antibiogram of VEB-1 ESBL–producing *A. baumannii* strains was similar for 275 (95%) of the 290 probable cases; the level of resistance to aminoglycosides varied slightly. A synergy image, signature of the presence of an ESBL, could not be observed between clavulanic acid and cefepime or ceftazidime disks on a routine antibiogram (Figure 3A) unless cloxacillin-containing plates that inhibit cephalosporinase activity were used (Figure 3B). Alternatively, incubation of the antibiogram at room temperature enhanced the identification of the synergy image (Figure 3C and D). One strain isolated in the Alpes-Maritimes district, next to Nice, was also resistant to colistin. All strains remained susceptible to imipenem.

**Figure 4:**
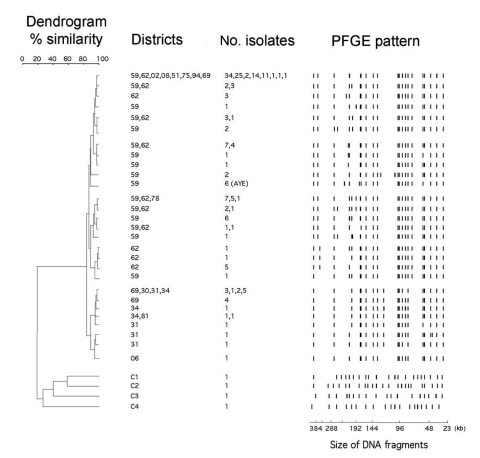
Digitized pulsed-field gel electrophoresis (PFGE) patterns and phylogenetic tree of 183 VEB-1–producing *Acinetobacter baumannii* isolates. Half of *A. baumannii* isolates from the 4 largest hospitals of 2 districts (59 and 62) were randomly selected over the entire epidemic period; all isolates from smaller hospitals or from other districts were included. The PFGE pattern of the *A. baumannii* AYE reference strain previously described is indicated in brackets (*16*). *Apa*I macrorestriction patterns were digitized and analyzed with Taxotron software (Institut Pasteur, Paris, France) to calculate Dice coefficients of correlation and to generate a dendrogram by the unweighted pair-group method using arithmetic averages clustering. The scale indicates the level of pattern similarity. PFGE results were interpreted according to the criteria of Tenover et al. (*25*). For a given PFGE pattern, the districts, along with the number of times a given PFGE pattern was found, are also indicated.

Of the 288 *A. baumannii* isolates, 275 (95%) contained the *bla*_VEB-1_ gene, according to PCR analysis, which shows a perfect correlation between the antibiogram and the PCR results. All but 2 notifying hospitals isolated the outbreak strain in their wards; for 1 hospital in Isère and 1 in Tarn, the isolate was not available for confirmatory testing. Genotyping by PFGE showed that all VEB-1 *A. baumannii* isolates were clonally related to each other and to the strain responsible for the 2001 outbreak (strain AYE [16]). Although most strains belonged to several subtypes of a given type, differing by only 1 or 2 bands, several strains were more distantly related and belonged to a different type. However, these isolates could be related to the main epidemic strains as illustrated on the dendrogram (Figure 4). The epidemic strain isolated in the southern part of the country differed from those in the northern part by at least 3 bands, and thus forms a separate cluster.

The sequence of *bla*_VEB-1_ gene and its genetic environment were identical to those previously published for *A. baumannii* strain AYE (*16**,**18*). Finally, the chromosomal position of the integron was verified for 20 isolates randomly chosen in the different regions.

### Control Measures

Recommendations for surveillance and control were disseminated to all hospitals (through specific postings on the InVS Web site and email from CCLIN to infection control units and laboratories) and then implemented by infection control units. Based on international guidelines (28, Table 2), the recommendations included the usual standard and contact precautions for limiting the spread of this pathogen within a hospital but added systematic screening in wards at high risk (e.g., intensive care units) and appropriate antimicrobial drug use. In addition, specific recommendations were dispatched to limit the spread of the strain from 1 hospital to another; hospitals were asked to report cases to local health departments and CCLIN, to limit patient admissions, and to inform other hospitals about infected or colonized patients before transferring them (2 hospitals even closed their wards to new patients).

**Table 2:** Recommendations for hospitals*†

Type	Recommendation
Notification	Report any case of infection or colonization with *Acinetobacter baumannii* strain showing resistance profile similar to that of 2001 outbreak strain (*16*) to CCLIN and local health department; attach copy of antibiogram.
Laboratory guidelines	Establish identification criteria based on antibiogram
	Store strains and contact central laboratory for microbiologic investigation
Medical wards	Inform all medical teams and paramedics of presence of bacterial strain
	Ensure appropriate use of antimicrobial drugs in high-risk wards
Infection control team	Set up systematic screening of patients in high-risk wards
	Reinforce isolation protocols and standard hygiene precautions throughout hospital
	Reinforce surface cleaning procedures in wards where infected or colonized patients have been
Patient transfer	Limit internal and external patient transfers
	Inform receiving hospitals of status of patients colonized or infected with ESBL-producing *A. baumannii*

*CCLIN, regional infection control coordinating centers; ESBL, extended-spectrum β-lactamase. †At the national level, recommendations were disseminated by the National Institute of Public Health (Institut de Veille Sanitaire) and the French Nosocomial Infection Early Warning, Investigation and Surveillance Network (Réseau d'Alerte, d'Investigation et de Surveillance des Infections Nosocomiales) through its website (http://www.invs.sante.fr/raisin, "alerte" section). At the regional level, they were disseminated to all hospital laboratories and infection control units by CCLIN through email notifications and their respective websites. In each hospital, they were implemented in the wards by infection control units assisted by CCLIN, when necessary.

## Discussion

This is the first report of a clonal ESBL-producing *A. baumannii* outbreak that was nationwide. It was traced in 53 hospitals, initially in northern France and later in 4 distant regions. Recognition of the outbreak and effective tracing of new cases was facilitated by several factors. First, in August 2001, a national nosocomial infection notification system based on specific reporting criteria, including unusual antimicrobial drug resistance profiles, was implemented in France (*23*). The system relies on hospital infection control units and is coordinated at the regional level by CCLIN created in 1992 (*22*) and at the national level by InVS, which enables events of national importance to be detected. Second, the French healthcare system is organized around large public, university, or regional tertiary care hospitals that serve an entire region. These hospitals include medical microbiology laboratories that are well connected to other laboratories in smaller public hospitals, therefore enabling prompt dissemination of laboratory guidelines. Third, the outbreak strain had a unique susceptibility profile that enabled effective screening of *A. baumannii* strains in hospital laboratories and referral to a central laboratory for confirmatory testing. This laboratory provided immediate feedback (<48 hours after strains were received) to hospitals, which facilitated prompt adaptation of local control measures, and to CCLIN and InVS, which enabled regular tracing of the strain dissemination and adaptation of recommendations.

Two factors make emergence of panresistant isolates through mutations in porins (*11*) or acquisition of plasmid-encoded carbapenemases (*8*), such as the *bla*_OXA-58_ gene (*28*); a concern. First, there may be no option but to treat patients infected with *A. baumannii*, particularly in the intensive care setting, because *A. baumannii* infection is associated with a higher case-fatality ratio in critically ill patients (*3**,**4**,**29**,**30*). Second, carbapenems were the last molecules active against *A. baumannii* VEB-1 isolates. Antimicrobial drug susceptibility of *A. baumannii* VEB-1 isolates remained relatively stable; all tested strains were fully susceptible to carbapenems and all but 1 was susceptible to colistin. The slight variations in their aminoglycoside susceptibilities reflected presumably different antimicrobial drug selection pressures in some of the hospitals.

Early warning and investigations of reported cases alerted all hospitals of the need for rapid identification and reporting of cases. Furthermore, early warning enabled timely assistance for implementing effective control measures. These investigations showed that the epidemic clone was already endemic in some hospitals, which suggests that once the strain is introduced into a hospital, eradicating it may be difficult. Early recognition of its presence and prompt implementation of strict infection control measures are therefore necessary to prevent its further spread and establishment of endemicity. Moreover, when several hospitals in the same network are affected by the same clone, coordinated measures must be implemented to effectively reduce its spread, as demonstrated for other multidrug-resistant bacteria such as vancomycin-resistant enterococci (*31*).

In this national investigation, spread within hospitals was not explored because it has been well described in the literature (*3**,**4**,**26**,**27*). While we reinforced the implementation of standard and contact precautions in affected hospitals, we recommended that other hospitals be informed (by flagged records) when infected or colonized patients are transferred to them. In some hospitals, fast-spreading clusters associated with deaths led to the closure of intensive care units; however, the effectiveness of such a measure needs further evaluation because transfer of these patients enhances the dissemination of the strain (*4*). In other hospitals, the prompt and strict application of barrier precautions, without closure of the intensive care unit, effectively controlled the outbreak.

Clones may emerge at different locations by independent selection of genetically related, circulating strains in the community or environment as a result of antibiotic use in hospitals (*2**,**8**,**32**,**33*). Otherwise, similar isolates may appear at different locations simply through direct spread from 1 hospital to another. Results of this investigation suggest that regional spread of the organism was mediated by patient transfers within regional healthcare networks. However, the appearance of the organism in hospitals in southern France, Lyon (central France), and Paris is difficult to explain by simple spread. Although *A. baumannii* isolates from southern and northern France were found in Lyon, no epidemiologic link could be found, and one cannot be sure that the cases in southern France were acquired from contact with patients or hospitals in northern France. Case reporting and recognition of the outbreak could have been delayed, and the epidemic situation that we observed might be the consequence of a spread that started earlier than 2003, even earlier than the 2001 outbreak in Valenciennes (*4*). Several facts support this hypothesis: 1) *bla*_VEB-1_ gene was characterized in 1996 in an *E. coli* strain isolated from a Vietnamese child hospitalized in France (*18*) and then in 2001 in an *A. baumannii* strain (*16*); 2) the 2001 *A. baumannii* isolates were clonally related, but at least 2 PFGE types had already been observed (*4*); 3) retrospective surveys recently conducted in a few large university hospitals in southern and northern France showed that *A. baumannii* VEB-1 isolates were present as early as January 2001 (data not shown). Finally, an alternative explanation could be that the isolates from northern and southern France were introduced separately into the country from an unknown common source.

The origin of this clone remains unknown because *A. baumannii* VEB-1 isolates have never been reported in other countries. All isolates were epidemiologically related, and most of them were similar enough to be considered as belonging to the same strain. However, the diversity of the total set of isolates was slightly greater than what is usually recovered in single-hospital outbreaks. Clonal diffusion with several pulsotypes has already been observed in the first *A. baumannii* VEB-1 outbreak described in 2001 (*4*). That isolates with indistinguishable PFGE profiles were found in many of the hospitals and that all isolates had similar profiles suggest that they could be considered relatively new, compared with older strains circulating in Europe (*32*). Although the origin of *bla*_VEB-1_ gene is presumably countries in Southeast Asia, it would be interesting to investigate the occurrence of such strains in these countries. Alternatively, a European *A. baumannii* strain might have acquired foreign DNA containing *bla*_VEB-1_ through either conjugation or transformation (*34*). Several multidrug-resistant *A. baumannii* strains have been found to be widespread in Europe (*32**,**33*), and epidemic carbapenem-resistant strains have been reported worldwide (*9**,**35**–**37*).

This report used data only from mandatory notifications and isolates received by the central laboratory; the full extent of this clone in France remains unknown. The large media coverage of this outbreak in late December 2003 may have discouraged hospitals from reporting cases; several isolates were actually sent to the central laboratory without being reported. Moreover, because mandatory notification is not patient-based, contact tracing of each patient could not be systematically performed. Data from notifications (date of first and last case, name of transferring facility for imported cases) enabled us to establish only when an index patient was admitted from a previously affected facility.

Because European countries were informed about this outbreak through the EU Early Warning and Response System, Belgian public health authorities were able to detect early and control a cluster of 3 cases in a nursing home close to the border with France (*38*). This clone should therefore be considered as an issue affecting hospitals not only in France but also in bordering countries, and this situation underlines the importance of supranational information exchange for early warning of antimicrobial drug resistance.

## Conclusion

The emergence and spread of this strain of VEB-1–producing *A. baumannii* isolates are worrisome and reflect the magnitude of antimicrobial drug resistance in France. Most of the reported cases occurred in northern France. The weekly number of reported cases dropped substantially after January 2004, which suggests that infection control recommendations were effective. After the alert was stopped in June 2004, only 12 notifications were received by InVS until December 2005; these notifications came from 8 hospitals (5 of them already known) and indicated a total of 17 VEB-1–producing *A. baumannii* infections. The last notification was received in May 2005, suggesting that the national outbreak was controlled. However, after June 2004 the notification system returned to its baseline setting; i.e., only multidrug-resistant *A. baumannii* infections were to be reported. Although we are confident that the national alert drastically helped reduce the clinical effect of the outbreak, we cannot rule out that the strain is still spread by colonized patients.

This study emphasizes the importance of an early warning network comprising infection control units and regional (CCLIN, health departments) and central (InVS and expert laboratory) structures. The results underscore the need for anticipating future, emerging antimicrobial drug resistance threats by combining laboratory and epidemiologic expertise. Early detection of emerging resistance mechanisms usefully completes surveillance data, which monitor the level of resistance but may miss the emergence of new phenomena.
